# Molecular responses and chromosomal aberrations in patients with polycythemia vera treated with peg-proline-interferon alpha-2b

**DOI:** 10.1002/ajh.23928

**Published:** 2015-03-02

**Authors:** Nicole CC Them, Klaudia Bagienski, Tiina Berg, Bettina Gisslinger, Martin Schalling, Doris Chen, Veronika Buxhofer-Ausch, Josef Thaler, Ernst Schloegl, Guenther A Gastl, Dominik Wolf, Karin Strecker, Alexander Egle, Thomas Melchardt, Sonja Burgstaller, Ella Willenbacher, Oleh Zagrijtschuk, Christoph Klade, Richard Greil, Heinz Gisslinger, Robert Kralovics

**Affiliations:** 1 CeMM Research Center for Molecular Medicine of the Austrian Academy of SciencesVienna, Austria; 2 Department of Internal Medicine I, Division of Hematology and Blood Coagulation, Medical University of ViennaVienna, Austria; 3 2nd Medical Department, Sozialmedizinisches Zentrum Ost—DonauspitalVienna, Austria; 4 Interne 1 Hemato-Oncology, Krankenhaus Der Elisabethinen LinzLinz, Austria; 5 Department of Internal Medicine IV, Wels-Grieskirchen HospitalWels, Austria; 6 Third Medical Department, Hanusch HospitalVienna, Austria; 7 Department of Internal Medicine V, Haematology & Oncology, Innsbruck Medical UniversityInnsbruck, Austria; 8 Medical Clinic III, Oncology, Hematology and Rheumatology, University Clinic of Bonn (UKB)Bonn, Germany; 9 Laboratory for Immunological and Molecular Cancer Research, Department of Internal Medicine III with Hematology, Medical Oncology, Hemostaseology, Infectious Diseases, Rheumatology, Oncologic Center, Paracelsus Medical UniversitySalzburg, Austria; 10 AOP Orphan Pharmaceuticals AGVienna, Austria

## Abstract

Fifty-one polycythemia vera (PV) patients were enrolled in the phase I/II clinical study PEGINVERA to receive a new formulation of pegylated interferon alpha (peg-proline-IFNα-2b, AOP2014/P1101). Peg-proline-IFNα-2b treatment led to high response rates on both hematologic and molecular levels. Hematologic and molecular responses were achieved for 46 and 18 patients (90 and 35% of the whole cohort), respectively. Although interferon alpha (IFNα) is known to be an effective antineoplastic therapy for a long time, it is currently not well understood which genetic alterations influence therapeutic outcomes. Apart from somatic changes in specific genes, large chromosomal aberrations could impact responses to IFNα. Therefore, we evaluated the interplay of cytogenetic changes and IFNα responses in the PEGINVERA cohort. We performed high-resolution SNP microarrays to analyze chromosomal aberrations prior and during peg-proline-IFNα-2b therapy. Similar numbers and types of chromosomal aberrations in responding and non-responding patients were observed, suggesting that peg-proline-IFNα-2b responses are achieved independently of chromosomal aberrations. Furthermore, complete cytogenetic remissions were accomplished in three patients, of which two showed more than one chromosomal aberration. These results imply that peg-proline-IFNα-2b therapy is an effective drug for PV patients, possibly including patients with complex cytogenetic changes. Am. J. Hematol. 90:288–294, 2015. © 2014 The Authors. American Journal of Hematology published by Wiley Periodicals, Inc.

## Introduction

Polycythemia vera (PV) represents a frequent type of BCR-ABL negative myeloproliferative neoplasm (MPN) and is characterized by an elevated erythrocyte mass, as well as a variable presence of thrombocytosis and leukocytosis 1,2. PV patients have a risk of developing thrombosis, bleeding and disease transformation to secondary myelofibrosis as well as secondary acute myeloid leukemia. In the vast majority (∼95%) of PV patients the *JAK2*-V617F mutation is found and in around 3% of PV patients the less common *JAK2* exon 12 mutations 3–9. The functional consequences of these mutations are a constitutively active JAK2 kinase signaling and cytokine hypersensitivity of myeloid cells 5,8,10–12.

Present treatment options for PV patients include phlebotomy, low-dose aspirin, hydroxyurea and interferon alpha (IFNα) 2. Already in the 1980s therapeutic efficacy of recombinant IFNα for MPN patients was reported 13–15. IFNα not only induced hematologic and molecular responses in most MPN patients but was also described to be nonleukemogenic 16. However, the use of recombinant IFNα was limited by frequent drug-related toxicities and subsequent treatment discontinuations. The chemical linkage of polyethylene glycol (peg) to IFNα increased the plasma half-life and improved the toxicity profile of peg-IFNα considerably 17. To date, the cellular and molecular mechanisms involved in the myelosuppressive effect of IFNα remain as unclear as the influence of constitutive and acquired genetic changes on the magnitude of IFNα responses. To evaluate the impact of acquired chromosomal aberrations we analyzed chromosomal lesions by high-resolution SNP microarrays in PV patients treated with a new formulation of peg-IFNα (AOP2014/P1101, peg-proline-IFNα-2b) in a phase I/II clinical trial.

## Methods

### Patient samples

Patients were enrolled in the phase I/II clinical study PEGINVERA (identifier: NCT01193699; www.clinicaltrials.gov) 18,19. The study was approved by the Institutional Review Board of the Medical University of Vienna and the Austrian Drug Agency (AGES) and all patients provided written consent. Granulocytes were isolated from peripheral blood by standard gradient centrifugation using Histopaque 1077 (Sigma–Aldrich, St. Louis, MO). Genomic DNA was isolated from granulocytes and whole blood samples using the Wizard Genomic DNA Purification Kit and the Maxwell 16 Tissue DNA Purification Kit (Promega, Madison, WI).

### Hematologic response criteria

Hematologic response was defined similar to the European LeukemiaNet (ELN) criteria 20. Complete hematologic response (CHR) required hematocrit <45%, platelet count ≤400 × 10^9^/L, white blood cell count ≤10 × 10^9^/L, normal spleen size, freedom of phlebotomy in the past 2 months and absence of thromboembolic events. In case of concomitant hydroxyurea (HU), at least 2 weeks after the last HU administration had to pass to qualify for diagnosing CHR. Partial hematologic response (PHR) was defined by hematocrit <45% without phlebotomy with persistent splenomegaly or elevated platelet count (>400 × 10^9^/L) or at least a 50% reduction of phlebotomy requirements. No hematologic response (NHR) was any response that did not fulfill CHR or PHR criteria. Best individual hematologic response was defined as the best response observed for a given patient. Detailed clinical outcomes were reported recently 18,19.

### Molecular response criteria

To evaluate molecular response, *JAK2*-V617F burden was determined by allele specific PCR and *JAK2* exon 12 mutations by a fragment analysis-based assay, as previously reported (each with a detection limit of 1%) 21–23. Molecular response was defined similar to the revised ELN criteria 24. Patients required a *JAK2* mutant allele burden of ≥20% at baseline for response assessment and latest follow-up samples were used for response definition. Complete molecular response (CMR) was defined by an undetectable residual *JAK2* mutant allele burden. Partial molecular response (PMR) required **≥**50% decrease in *JAK2* mutant allele burden from baseline value and no molecular response (NMR) was a <50% reduction in *JAK2* mutant allele burden from baseline value.

### SNP microarray analysis

DNA samples were processed and hybridized to Genome Wide Human SNP 6.0 arrays (Affymetrix, Santa Clara, CA) according to the manufacturer’s protocol. Raw data was analyzed for quality, copy number variation and loss of heterozygosity using Genotyping Console version 4.1.1 software (Affymetrix). Chromosomal aberrations (deletions, gains and uniparental disomies-UPDs) were annotated manually. UPDs were annotated if they had a terminal location (end of chromosomal arm) and size of ≥1 Mb. Terminal UPDs in patients with extensive (>10 Mb) interstitial runs of homozygosity and all aberrations that mapped to known copy number variation loci according to the Database of Genomic Variants (DGV version 10, human reference genome assembly hg19) were excluded.

### Statistical analysis

To test for significant differences Fisher’s exact test was used for categorical data and Mann–Whitney test for continuous data. Wilcoxon signed rank test was used to compare *JAK2* mutant allele burden at baseline and on peg-proline-IFNα-2b treatment. All tests were two-tailed and *P* values lower than 0.05 were considered significant. For data management and analysis Microsoft Office Excel 2007 (Microsoft, Redmond, WA) and GraphPad Prism version 6.01 for Windows (GraphPad Software, La Jolla, CA) were used.

## Results

### Patient characteristics

Fifty-one PV patients were included in the phase I/II clinical trial PEGINVERA to receive a new formulation of peg-IFNα (AOP2014/P1101, peg-proline-IFNα-2b). Patient characteristics are outlined in Table 1 and Supporting Information Table I. The cohort included 31 male patients (61%) and the median age of patients was 56 years. The median time from PV diagnosis to study entry was 511 days and 17 patients (33%) received hydroxyurea before study entry. For 11 patients (22%) PV related events (thrombosis and bleeding) were observed before investigational treatment. Patients received a median dose of 244 μg peg-proline-IFNα-2b and the median follow-up time of the cohort was 80 weeks. The median *JAK2* mutant allele burden at treatment start was 41% and *JAK2* mutant allele burden changes over time for the whole cohort are displayed in Supporting Information Fig. 1.

**Table 1:** Clinical Characteristics of PV Patients Treated With Peg-proline-IFNα-2b

Clinical characteristics	Number	% or range
Total no. of patients	51	
Sex, male	31	61
Median age (years)	56	35–82
Median days, PV diagnosis to study entry	511	0–7245
Hydroxyurea pretreatment, patients	17	33
PV related event before investigational treatment, patients	11	22
Median dose peg-proline-IFNα-2b (µg)	244	56–540
Median follow-up time (weeks)	80	2–174
Median baseline *JAK2* mutant allele burden (%)	41	0–99

Baseline, peg-proline-IFNα-2b treatment start; PV related event, thrombosis, and bleeding.

### Influence of chromosomal aberrations on peg-proline-IFNα-2b response

**Figure 1:**
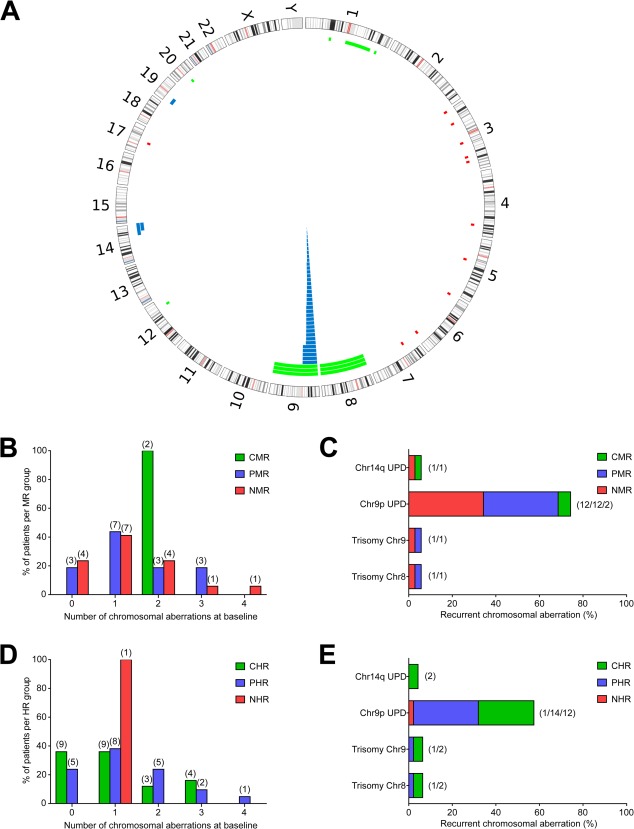
Response to peg-proline-IFNα-2b can be achieved independently of chromosomal aberrations. A: Circos plot depicting observed chromosomal aberrations, detected by affymetrix Genome-wide human SNP 6.0 arrays, for 51 PV patients at baseline. (bars, physical position, and size of aberrations—for better visibility, 3.5 mb were added to aberrations <6.9 mb; green, gains; red, deletions; blue, UPDs). B: Distribution of the number of chromosomal aberrations at baseline in the three molecular response groups. C: Recurrent chromosomal aberrations (present in ≥2 patients) at baseline for different molecular responding PV patients treated with peg-proline-IFNα-2b. D: Distribution of the number of chromosomal aberrations at baseline in the three hematologic response groups. E: Recurrent chromosomal aberrations (present in ≥2 patients) at baseline for different hematologic responding PV patients treated with peg-proline-IFNα-2b. (PV, polycythemia vera; baseline, peg-proline-IFNα-2b treatment start; UPD, uniparental disomy; chr, chromosome; CMR, complete molecular response; PMR, partial molecular response; NMR, no molecular response; CHR, complete hematologic response; PHR, partial hematologic response; NHR, no hematologic response; numbers in brackets show the actual numbers of patients).

To analyze chromosomal aberrations for patients treated with peg-proline-IFNα-2b, we performed high-resolution SNP microarrays for 51 baseline samples (at start of peg-proline-IFNα-2b treatment) and 46 follow-up samples (latest follow-up samples that were available at time of array analysis) (Supporting Information Table II and Fig. 1A). Typical aberrations for MPN were observed, such as chromosome 9p uniparental disomies (UPDs), 14q UPDs, trisomies of chromosome 8 and 9. Over 70% of the baseline samples showed at least one chromosomal aberration, of which chromosome 9p UPDs were the most prevalent ones.

To evaluate if cytogenetic lesions influence the response to peg-proline-IFNα-2b we compared the number of chromosomal aberrations in the different response groups. Similar numbers of chromosomal aberrations were observed in the diverse molecular response groups (Fig. 1B). Also in hematologic response groups no differences were found (Fig. 1D). Furthermore, we assessed if specific, recurrent (present in at least two patients) cytogenetic changes might explain the diverse response outcomes. Lesions that were present in more than one patient were distributed over all three molecular response groups with no significant differences in their frequencies (Fig. 1C). Comparing hematologic response groups revealed similar results with no associations of recurrent aberrations to specific response groups and both chromosome 14q UPDs were observed in complete hematologic response (CHR) patients (Fig. 1E). Overall this indicates that responses on molecular and hematologic levels are achieved independently of chromosomal aberrations.

### Peg-proline-IFNα-2b induces hematologic and molecular response

**Figure 2:**
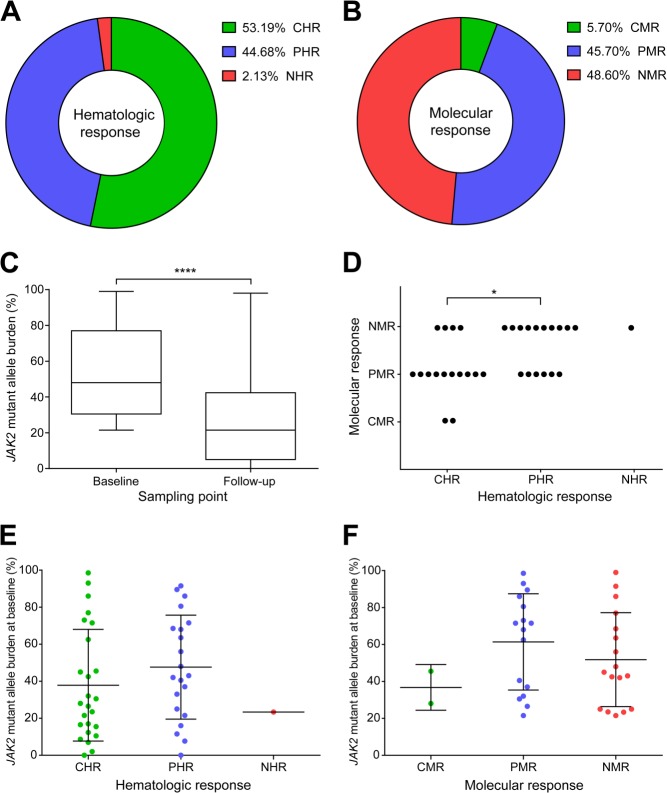
High response rates to peg-proline-IFNα-2b, that are likely not influenced by the *JAK2* mutant allele burden at treatment start. A: Best individual hematologic response, based on clinical parameters, for PV patients treated with peg-proline-IFNα-2b. B: Molecular response, based on *JAK2* mutant allele burden decrease at latest follow-up sample, for PV patients treated with peg-proline-IFNα-2b. C: Significant overall *JAK2* mutant allele burden decrease for PV patients treated with peg-proline-IFNα-2b at the current median follow-up time of 19 month. (horizontal lines, median values; bars, minimum and maximum values; box, values between 25th and 75th percentile; *P* < 0.0001). D: Correlation of hematologic and molecular responses for PV patients treated with peg-proline-IFNα-2b. Significant better molecular responses for CHR patients than for patients with PHR (*P* – 0.0373). E: *JAK2* mutant allele burden at baseline for different hematologic response groups. (horizontal lines, mean values; bars, standard deviation). F: *JAK2* mutant allele burden at baseline for different molecular response groups. (horizontal lines, mean values; bars, standard deviation). (PV, polycythemia vera; CHR, complete hematologic response; PHR, partial hematologic response; NHR, no hematologic response; CMR, complete molecular response; PMR, partial molecular response; NMR, no molecular response; baseline, peg-proline-IFNα-2b treatment start).

From the 51 included patients 47 were evaluable for hematologic responses with a median follow-up time of 74 weeks (range, 10–146 weeks). A partial hematologic response (PHR) was present in 45% of the patients (Fig. 2A). Only one patient (2%) did not show any response (NHR) and 53% of the patients reached a CHR. The median time to CHR was 18 weeks (range, 10–122 weeks).

Thirty-five patients were assessed for molecular responses as they had a *JAK2* mutant allele burden of 20% or higher at treatment start and at least one follow-up sample available (Supporting Information Table III). The median follow-up time for molecular response assessment was 584 days (range, 61–1,127 days). A molecular response was generally accomplished gradually over time and the median time to response was 433 days (range, 133–633 days). Molecular responses were achieved in 51% of the patients and in 26% a residual *JAK2* mutant allele burden of 5% or lower was accomplished (Fig. 2B). In two patients (6%) the *JAK2*-V617F mutation was undetectable in the latest follow-up sample, indicating a complete molecular response (CMR). A partial molecular response (PMR) required a decrease of 50% or more of the *JAK2* mutant allele burden. This was accomplished in 46% of the patients (Fig. 2B). As PMR patients showed a continuous reduction of the *JAK2* mutant allele burden, we suspect that the remission depth will further improve over time on continuous therapy (Supporting Information Table III). Moreover, the overall *JAK2* mutant allele burden of the 35 patients analyzed for molecular response decreased significantly on peg-proline-IFNα-2b treatment (*P* < 0.0001; Fig. 2C). The *JAK2* mutant allele burden changed from a median of 48% at treatment start to 21.5% at the current median follow-up time of 19 month. We have not observed patients that initially showed a molecular response and relapsed molecularly during therapy to no molecular response (NMR).

Next we compared the hematologic and molecular responses in 33 patients that had both response data available (Fig. 2D). Patients achieving CHR showed significantly better molecular responses than PHR patients (*P* – 0.0373). Notably, in some cases a CHR or PHR was achieved without a molecular response. In conclusion, peg-proline-IFNα-2b leads to a sound clinical and molecular response in the majority of PV patients.

### Factors affecting the response to peg-proline-IFNα-2b

Because the number and type of chromosomal aberrations had no significant influence on the outcome of the therapy, we next assessed which other parameters could have affected response rates. The first factor examined was the *JAK2* mutant allele burden at the start of therapy. Both clinical and molecular responses were achieved independently of the initial *JAK2* mutant allele burden (Fig. 2E,F). Furthermore, we divided patients into two groups according to their *JAK2* mutant allele burden at baseline (low, 20–59%; high ≥60%). We observed similar median times to molecular response in both groups (442 and 423 days, respectively). Thus, *JAK2* mutant allele burden at treatment start seems to have no impact on peg-proline-IFNα-2b treatment outcomes, at least in our cohort. To exclude observation time biases we focused our analysis on patients with a follow-up time of more than 400 days (mean duration to achieve a molecular response). No significant differences between molecular responding (CMR and PMR) and nonresponding (NMR) patients were seen for factors such as sex, age or previous hydroxyurea therapy, besides a trend for older patients responding worse to the therapy (Table 2). Overall these results suggest that other factors (such as germline variants) might influence the response to peg-proline-IFNα-2b.

**Table 2:** Factors Influencing Molecular Response to Peg-proline-IFNα-2b

Factor	Molecular responders (*n* – 15)	Molecular nonresponders (*n* – 7)	*P* value
Median age (years)	55	69	0.0577
Median number of chromosomal aberrations	1	1	0.2064
Sex, female, *n* (%)	1 (7%)	2 (29%)	0.2273
Hydroxyurea pretreatment, *n* (%)	4 (27%)	4 (57%)	0.3426
Median dose peg-proline-IFNα-2b (µg)	261	279	0.6273
Median follow-up time for molecular response (days)	861	713	0.7794
Median baseline *JAK2* mutant allele burden (%)	63	64	0.8493
Median days, PV diagnosis to study entry	511	428	0.8784
Recurrent aberration (≥2 patients)			
Chr9p UPD, *n* (%)	11 (73%)	5 (71%)	1.0000
Chr14q UPD, *n* (%)	1 (7%)	1 (14%)	1.0000

Patients with follow up time >400 days were analyzed; molecular responders, partial and complete molecular response; baseline, peg-proline-IFNα-2b treatment start; Chr, chromosome; UPD, uniparental disomy.

### Cytogenetic changes during peg-proline-IFNα-2b therapy

Chromosomal aberrations that were not present at peg-proline-IFNα-2b treatment start and only found in the follow-up sample may indicate clonal evolution during therapy. These emerging clones might cause acquired peg-proline-IFNα-2b resistance and/or accelerated disease progression. Acquired cytogenetic lesions were detected in three patients. Patient 5007 with PMR showed a small clone with a deletion of chromosome Y, patient 1004 with NMR had a single gene deletion on chromosome 10p (*USP6NL*) and patient 3003 (baseline *JAK2* mutant allele burden lower than 20%) showed a single gene gain on chromosome 3q (*FXR1*) and a single gene deletion on chromosome 7p (*NXPH1*) (Supporting Information Table II).

**Figure 3:**
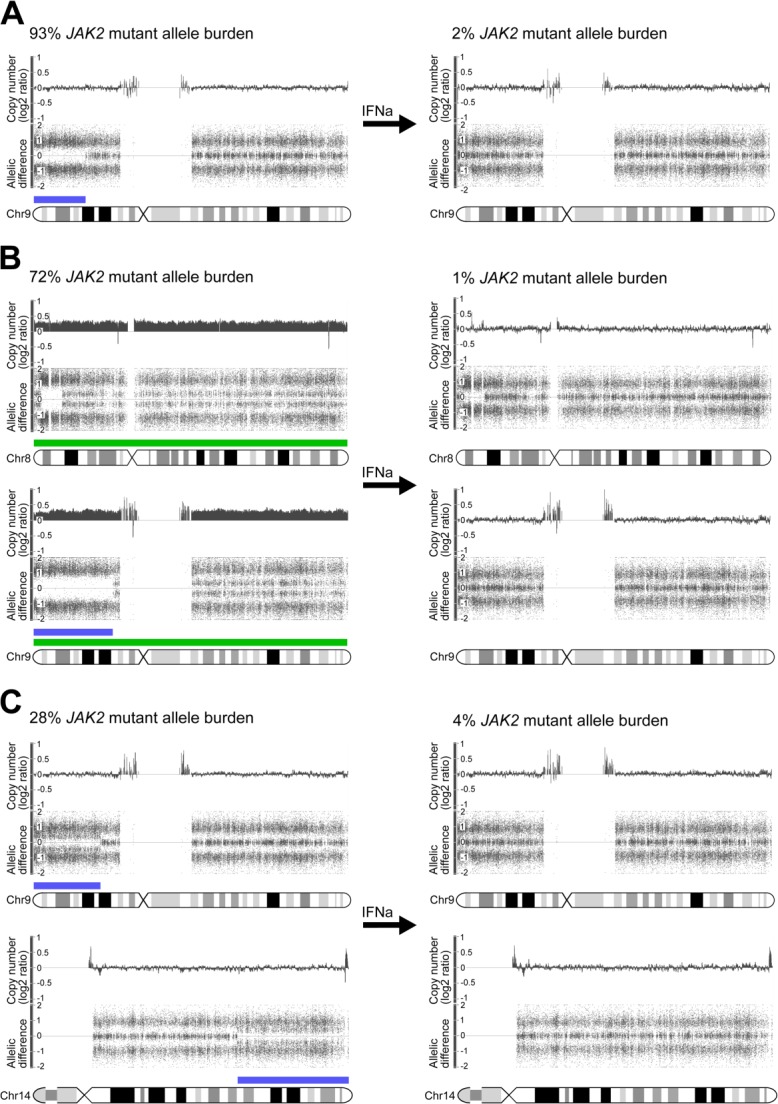
Complete cytogenetic remission in 3 PV patients treated with peg-proline-IFNα-2b. Plots represent data from affymetrix Genome-wide human SNP 6.0 arrays, analyzed with the affymetrix genotyping console version 4.1.1 software for chromosomal aberrations (copy number represented as log2 ratio to normal samples and allelic difference, where an allelic difference of 0 displays a heterozygous status). Horizontal bars represent physical position and size of chromosomal aberrations (in green, gains and blue, UPDs). Plots on the left show chromosomal lesions and the *JAK2* mutant allele burden at peg-proline-IFNα-2b treatment start and on the right on treatment. A: Patient 2002 showed at treatment start a Chr9p UPD, which was lost on peg-proline-IFNα-2b treatment. B: Patient 4010 showed at treatment start a Chr9p UPD, trisomies of Chr8 and Chr9, which were lost on peg-proline-IFNα-2b treatment. C: Patient 5010 showed at treatment start a Chr9p and Chr14q UPD, which were lost on peg-proline-IFNα-2b treatment. (PV, polycythemia vera; UPD, uniparental disomy; chr, chromosome; IFNa, peg-proline-IFNα-2b).

Chromosomal aberrations that are lost on peg-proline-IFNα-2b treatment indicate complete cytogenetic remission, thus a molecular response. In three patients such a complete cytogenetic response was achieved, all three of them showing chromosome 9p UPDs at treatment start (Fig. 3). One patient had in addition to the 9p UPD trisomies of chromosome 8 and 9 and another one a chromosome 14q UPD. This indicates that peg-proline-IFNα-2b is able to target clones with lesions in addition to mutated *JAK2*.

## Discussion

In a previous study we reported that chromosomal aberrations emerged at the time of IFNα resistance in a patient with primary myelofibrosis 25. Therefore, we hypothesized that either specific types or the number of cytogenetic lesions might impact IFNα treatment outcome. In this study we performed a detailed analysis of molecular responses to peg-proline-IFNα-2b in a large cohort of PV patients in which high-resolution SNP array karyotypes were obtained. We observed similar numbers and types of chromosomal aberrations in the different response groups (Fig. 1B–E and Table 2). Thus, responses to peg-proline-IFNα-2b are independent of chromosomal lesions in patients with PV. Patients with complex karyotypes may perhaps equally benefit from peg-proline-IFNα-2b therapy as patients with no detectable chromosomal aberration.

Peg-proline-IFNα-2b treatment led to high response rates on both molecular and clinical levels and as previously reported, molecular responses were generally preceded by hematologic responses (Fig. 2A–C) 26,27. CHR was accompanied by significantly better responses on molecular levels compared to PHR (Fig. 2D). Similar results were reported for MPN patients that were treated with hydroxyurea, where the main factor for completing a major molecular response was a major hematologic response 28. In contrast, another study reported that responses on clinical and molecular levels were not associated with each other 29. We observed as well absence of molecular response for some CHR or PHR cases, but at a much lower rate.

No difference in the *JAK2* mutant allele burden was observed in the diverse response groups at treatment start, suggesting that the outcome of peg-proline-IFNα-2b therapy is likely independent of baseline *JAK2* mutant allele burden (Fig. 2E,F and Table 2). Similar observations were reported for peg-IFNα-2a treated PV patients, where the *JAK2*-V617F reduction was not influenced by the initial *JAK2*-V617F allele burden 26. Moreover, PV patients with a fast molecular response to peg-IFNα-2a showed no significant difference in their initial *JAK2*-V617F allele burden compared to slow molecular responders 30. Factors such as age and hydroxyurea pretreatment did not significantly influence the outcome to peg-proline-IFNα-2b therapy (Table 2). Complete cytogenetic remission on peg-proline-IFNα-2b treatment was observed in three patients, showing that clones with more lesions than *JAK2* mutation are also sensitive to the therapy (Fig. 3).

Because we did not find evidence for chromosomal lesions being responsible for an absence of IFNα response, the question on the mechanisms underlying IFNα resistance remains open. Recent studies report inconclusive findings whether clones with mutated *TET2* can persist IFNα therapy and potentially be involved in IFNα response outcomes 31–33. Also the frequency of MPN associated mutations in addition to *JAK2* did not significantly influence IFNα responses 33,34.

We did not observe any relapses to NMR, indicating that a resistance to IFNα was present from the start of therapy. This intrinsic resistance might be caused by somatic changes already present at treatment start or constitutive germline variants, such as in the case of hepatitis C therapy where *IL28B* was significantly associated with the response to IFNα and ribavirin 35–37. Because we and others could not obtain sufficient evidence for the involvement of somatic aberrations in IFNα resistance, we believe that germline variants may determine the outcome to IFNα in most cases. The observed high response rates, that are independent of chromosomal aberrations, makes peg-proline-IFNα-2b a promising therapeutic for PV. Future studies should help to reveal factors that affect IFNα therapy and ultimately identify resistance mechanisms as well as patients that benefit from IFNα therapy.
