# Supplementary material for: Molecular responses and chromosomal aberrations in patients with polycythemia vera treated with peg-proline-interferon alpha-2b
Source: Am J Hematol. 2015 Mar 2;90(4):288–94. doi: 10.1002/ajh.23928 (PMC4657499; doi:10.1002/ajh.23928)
Supplement: Supplementary file 1 [file ajh0090-0288-sd1.doc]

**Supporting Information for**

**Molecular responses and chromosomal aberrations in patients with polycythemia vera treated with peg-proline-interferon alpha-2b**

**Content**

**Supporting Information Table I.** Detailed clinical characteristics and hematologic responses in PV patients treated with peg-proline-IFNα-2b.

**Supporting Information Table II.** Detailed annotations of observed chromosomal aberrations.

**Supporting Information Table III.** *JAK2* mutant allele burden and molecular responses over time in PV patients treated with peg-proline-IFNα-2b.

**Supporting Information Figure 1**. *JAK2* mutant allele burden change over time for the complete cohort of PV patients treated with peg-proline-IFNα-2b.

| **Supporting Information Table I. Detailed clinical characteristics and hematologic responses in PV patients treated with peg-proline-IFNα-2b.** | | | | | | | | | |
| --- | --- | --- | --- | --- | --- | --- | --- | --- | --- |
|  |  |  |  |  |  |  |  |  |  |
| **Patient ID** | **Sex, male=1, female=2** | **Age, years** | **PV diagnosis to study entry, days** | **HU-pre-treatment, yes=1, no=0** | **Mean peg-proline-IFNα-2b dose, µg** | **PV related event (thrombosis, bleeding), yes=1, no=0** | **Follow-up time, weeks** | **Best individual hematological response, complete=1, partial=2, no=3** | **JAK2 mutant allele burden at peg-proline-IFNα-2b treatment start** |
| 1001 | 1 | 55 | 2108 | 1 | 299 | 0 | 167 | 2 | 42.00% |
| 1002 | 1 | 68 | 428 | 0 | 279 | 0 | 168 | 2 | 86.00% |
| 1003 | 2 | 55 | 1049 | 0 | 171 | 0 | 15 | 2 | 68.50% |
| 1004 | 1 | 80 | 5159 | 1 | 158 | 1 | 164 | 1 | 23.50% |
| 1005 | 2 | 52 | 2063 | 0 | 275 | 1 | 12 | 2 | 43.00% |
| 1008 | 1 | 68 | 191 | 0 | 198 | 0 | 150 | 1 | 15.50% |
| 1009 | 1 | 74 | 146 | 0 | 114 | 0 | 133 | 1 | 98.50% |
| 1010 | 2 | 63 | 4508 | 0 | 202 | 1 | 128 | 1 | 17.00% |
| 1011 | 1 | 51 | 406 | 0 | 151 | 0 | 130 | 2 | 89.50% |
| 1012 | 2 | 66 | 0 | 0 | 254 | 0 | 36 | 1 | 42.50% |
| 1013 | 2 | 60 | 1848 | 0 | 265 | 1 | 24 | 3 | 23.33% |
| 1014 | 1 | 65 | 1160 | 1 | 296 | 0 | 94 | 2 | 68.00% |
| 1015 | 1 | 60 | 0 | 0 | 195 | 0 | 86 | 2 | 91.50% |
| 1016 | 2 | 48 | 420 | 0 | 232 | 1 | 54 | 1 | 10.50% |
| 1017 | 1 | 43 | 88 | 0 | 90 | 0 | 71 | 2 | 16.00% |
| 1018 | 1 | 71 | 7245 | 0 | 249 | 1 | 51 | 2 | 56.00% |
| 1020 | 2 | 54 | 1261 | 0 | 85 | 1 | 25 | 2 | 25.00% |
| 1021 | 2 | 78 | 0 | 0 | 131 | 0 | 49 | 2 | 21.50% |
| 1022 | 1 | 41 | 3245 | 0 | 169 | 0 | 106 | 2 | 71.50% |
| 2001 | 1 | 51 | 0 | 0 | 270 | 0 | 174 | 1 | 30.50% |
| 2002 | 1 | 55 | 71 | 1 | 268 | 0 | 155 | 1 | 93.00% |
| 2003 | 1 | 63 | 5097 | 1 | 225 | 1 | 4 | not available | 99.00% |
| 2005 | 1 | 53 | 770 | 1 | 435 | 0 | 142 | 1 | 12.33% |
| 3001 | 1 | 50 | 2338 | 1 | 353 | 0 | 148 | 1 | 45.50% |
| 3002 | 1 | 62 | 2029 | 1 | 308 | 0 | 140 | 1 | 7.00% |
| 3003 | 2 | 44 | 274 | 1 | 540 | 0 | 16 | 1 | 8.67% |
| 3004 | 2 | 54 | 737 | 1 | 300 | 0 | 18 | 1 | 0.00% |
| 3005 | 1 | 47 | 0 | 0 | 363 | 1 | 109 | 1 | 86.00% |
| 3006 | 1 | 56 | 0 | 0 | 300 | 0 | 32 | 1 | 32.00% |
| 3007 | 2 | 69 | 735 | 1 | 244 | 0 | 6 | not available | 25.00% |
| 4004 | 1 | 42 | 0 | 0 | 150 | 0 | 2 | not available | 48.50% |
| 4006 | 2 | 48 | 331 | 1 | 315 | 0 | 58 | 2 | 33.00% |
| 4007 | 2 | 58 | 794 | 0 | 425 | 0 | 58 | 2 | 48.00% |
| 4009 | 2 | 59 | 173 | 0 | 93 | 0 | 35 | 2 | 0.00% |
| 4010 | 2 | 47 | 239 | 0 | 224 | 0 | 54 | 1 | 71.50% |
| 5001 | 1 | 56 | 209 | 1 | 210 | 0 | 30 | 2 | 7.67% |
| 5002 | 1 | 66 | 511 | 1 | 170 | 0 | 164 | 1 | 73.00% |
| 5003 | 2 | 53 | 648 | 0 | 261 | 0 | 55 | 1 | 21.50% |
| 5004 | 1 | 43 | 246 | 0 | 242 | 0 | 136 | 1 | 16.50% |
| 5005 | 1 | 71 | 2174 | 0 | 235 | 0 | 160 | 1 | 26.50% |
| 5007 | 1 | 69 | 0 | 0 | 181 | 0 | 129 | 1 | 62.50% |
| 5008 | 1 | 35 | 5210 | 0 | 289 | 0 | 58 | 2 | 40.50% |
| 5010 | 1 | 76 | 0 | 0 | 172 | 0 | 80 | 1 | 28.00% |
| 5011 | 1 | 55 | 1865 | 0 | 178 | 0 | 74 | 2 | 37.00% |
| 5012 | 1 | 55 | 108 | 0 | 347 | 0 | 54 | 1 | 2.00% |
| 6001 | 2 | 82 | 0 | 0 | 56 | 1 | 96 | 1 | 45.00% |
| 6003 | 1 | 64 | 2901 | 0 | 414 | 0 | 162 | 2 | 80.50% |
| 6004 | 2 | 40 | 1006 | 0 | 515 | 0 | 129 | 2 | 11.50% |
| 6005 | 2 | 70 | 2542 | 1 | 422 | 0 | 117 | 2 | 63.50% |
| 6006 | 1 | 69 | 275 | 1 | 427 | 1 | 104 | 1 | 77.00% |
| 6011 | 2 | 78 | 3899 | 1 | 188 | 0 | 2 | not available | 77.50% |

| **Supporting Information Table II. Detailed annotations of observed chromosomal aberrations.** | | | | | | |
| --- | --- | --- | --- | --- | --- | --- |
|  |  |  |  |  |  |  |
| **Sample ID** | **Sampling point** | **Total no. of aberrations** | **Type of aberration** | **Chromosome** | **Start (bp)** | **End (bp)** |
| AOP_01001_GD | Baseline | 0 |  |  |  |  |
| AOP_01002_GD | Baseline | 1 | UPD | chr9 | 1 | 35980000 |
| AOP_01003_GD | Baseline | 1 | UPD | chr9 | 1 | 28250000 |
| AOP_01004_GD | Baseline | 0 |  |  |  |  |
| AOP_01005_GD | Baseline | 2 | gain | chr8 | 1 | 146364022 |
| AOP_01005_GD | Baseline |  | gain | chr9 | 1 | 141213431 |
| AOP_01008_GD | Baseline | 1 | del | chr3 | 174940000 | 175010000 |
| AOP_01009_GD | Baseline | 1 | UPD | chr9 | 1 | 36730000 |
| AOP_01010_GD | Baseline | 0 |  |  |  |  |
| AOP_01011_GD | Baseline | 1 | UPD | chr9 | 1 | 28240000 |
| AOP_01012_GD | Baseline | 1 | UPD | chr9 | 1 | 30730000 |
| AOP_01013_WD | Baseline | 1 | UPD | chr9 | 1 | 32260000 |
| AOP_01014_GD | Baseline | 2 | UPD | chr9 | 1 | 20400000 |
| AOP_01014_GD | Baseline |  | del | chr7 | 39940000 | 40010000 |
| AOP_01015_WD | Baseline | 1 | UPD | chr9 | 1 | 24430000 |
| AOP_01016_WD | Baseline | 3 | gain | chr8 | 1 | 146364022 |
| AOP_01016_WD | Baseline |  | gain | chr9 | 1 | 141213431 |
| AOP_01016_WD | Baseline |  | del | chr3 | 5510000 | 6140000 |
| AOP_01017_WD | Baseline | 1 | del | chr3 | 114250000 | 114550000 |
| AOP_01018_WD | Baseline | 2 | UPD | chr9 | 1 | 12790000 |
| AOP_01018_WD | Baseline |  | UPD | chr9 | 1 | 32700000 |
| AOP_01020_WD | Baseline | 0 |  |  |  |  |
| AOP_01021_WD | Baseline | 4 | UPD | chr9 | 1 | 19440000 |
| AOP_01021_WD | Baseline |  | UPD | chr19 | 1 | 19690000 |
| AOP_01021_WD | Baseline |  | gain | chr1 | 223290000 | 223590000 |
| AOP_01021_WD | Baseline |  | del | chr3 | 159900000 | 160000000 |
| AOP_01022_WD | Baseline | 1 | UPD | chr9 | 1 | 33100000 |
| AOP_02001_GD | Baseline | 1 | gain | chr1 | 76130000 | 76730000 |
| AOP_02002_GD | Baseline | 1 | UPD | chr9 | 1 | 23310000 |
| AOP_02003_GD | Baseline | 2 | UPD | chr9 | 1 | 35480000 |
| AOP_02003_GD | Baseline |  | UPD | chr9 | 1 | 49000000 |
| AOP_02005_GD | Baseline | 0 |  |  |  |  |
| AOP_03001_GD | Baseline | 2 | UPD | chr9 | 1 | 49000000 |
| AOP_03001_GD | Baseline |  | gain | chr20 | 32810000 | 33080000 |
| AOP_03002_GD | Baseline | 0 |  |  |  |  |
| AOP_03003_GD | Baseline | 0 |  |  |  |  |
| AOP_03004_WD | Baseline | 0 |  |  |  |  |
| AOP_03005_GD | Baseline | 3 | UPD | chr9 | 1 | 35640000 |
| AOP_03005_GD | Baseline |  | del | chr4 | 165060000 | 165160000 |
| AOP_03005_GD | Baseline |  | del | chr17 | 33670000 | 33770000 |
| AOP_03006_GD | Baseline | 1 | UPD | chr9 | 1 | 49000000 |
| AOP_03007_WD | Baseline | 0 |  |  |  |  |
| AOP_04004_WD | Baseline | 1 | UPD | chr9 | 1 | 49000000 |
| AOP_04006_WD | Baseline | 1 | UPD | chr9 | 1 | 23170000 |
| AOP_04007_WD | Baseline | 3 | UPD | chr9 | 1 | 6980000 |
| AOP_04007_WD | Baseline |  | gain | chr1 | 125000001 | 205920000 |
| AOP_04007_WD | Baseline |  | del | chr6 | 161930000 | 162420000 |
| AOP_04009_WD | Baseline | 3 | UPD | chr9 | 1 | 37930000 |
| AOP_04009_WD | Baseline |  | del | chr5 | 75830000 | 75920000 |
| AOP_04009_WD | Baseline |  | del | chr6 | 5520000 | 5690000 |
| AOP_04010_WD | Baseline | 3 | UPD | chr9 | 1 | 35660000 |
| AOP_04010_WD | Baseline |  | gain | chr8 | 1 | 146364022 |
| AOP_04010_WD | Baseline |  | gain | chr9 | 1 | 141213431 |
| AOP_05001_GD | Baseline | 0 |  |  |  |  |
| AOP_05002_GD | Baseline | 3 | UPD | chr9 | 1 | 28200000 |
| AOP_05002_GD | Baseline |  | UPD | chr9 | 1 | 36370000 |
| AOP_05002_GD | Baseline |  | del | chr3 | 48010000 | 48090000 |
| AOP_05003_GD | Baseline | 0 |  |  |  |  |
| AOP_05004_GD | Baseline | 0 |  |  |  |  |
| AOP_05005_GD | Baseline | 0 |  |  |  |  |
| AOP_05007_GD | Baseline | 1 | UPD | chr9 | 1 | 36730000 |
| AOP_05008_GD | Baseline | 0 |  |  |  |  |
| AOP_05010_WD | Baseline | 2 | UPD | chr9 | 1 | 30160000 |
| AOP_05010_WD | Baseline |  | UPD | chr14 | 69390000 | 107349540 |
| AOP_05011_WD | Baseline | 2 | UPD | chr9 | 1 | 28790000 |
| AOP_05011_WD | Baseline |  | UPD | chr9 | 1 | 35980000 |
| AOP_05012_WD | Baseline | 1 | gain | chr12 | 103750000 | 104330000 |
| AOP_06001_GD | Baseline | 2 | UPD | chr9 | 1 | 28210000 |
| AOP_06001_GD | Baseline |  | UPD | chr14 | 81790000 | 107349540 |
| AOP_06003_GD | Baseline | 2 | UPD | chr9 | 1 | 20480000 |
| AOP_06003_GD | Baseline |  | UPD | chr9 | 1 | 35520000 |
| AOP_06004_GD | Baseline | 0 |  |  |  |  |
| AOP_06005_WD | Baseline | 1 | UPD | chr9 | 1 | 49000000 |
| AOP_06006_WD | Baseline | 1 | UPD | chr9 | 1 | 16000000 |
| AOP_06011_WD | Baseline | 1 | UPD | chr9 | 1 | 28260000 |
| AOP01001L_WD | Follow-up | 0 |  |  |  |  |
| AOP01002L_WD | Follow-up | 1 | UPD | chr9 | 1 | 35980000 |
| AOP01003B_GD | Follow-up | 1 | UPD | chr9 | 1 | 28250000 |
| AOP01004L_WD | Follow-up | 1 | del | chr10 | 11550000 | 11640000 |
| AOP01005B_GD | Follow-up | 2 | gain | chr8 | 1 | 146364022 |
| AOP01005B_GD | Follow-up |  | gain | chr9 | 1 | 141213431 |
| AOP01008I_WD | Follow-up | 1 | del | chr3 | 174940000 | 175010000 |
| AOP01009J_WD | Follow-up | 1 | UPD | chr9 | 1 | 36730000 |
| AOP01010H_WD | Follow-up | 0 |  |  |  |  |
| AOP01011J_WD | Follow-up | 1 | UPD | chr9 | 1 | 28240000 |
| AOP01012E_WD | Follow-up | 1 | UPD | chr9 | 1 | 30730000 |
| AOP01013B_WD | Follow-up | 1 | UPD | chr9 | 1 | 32260000 |
| AOP01014C_WD | Follow-up | 2 | UPD | chr9 | 1 | 20400000 |
| AOP01014C_WD | Follow-up |  | del | chr7 | 39940000 | 40010000 |
| AOP01015C_WD | Follow-up | 1 | UPD | chr9 | 1 | 24430000 |
| AOP01016C_WD | Follow-up | 3 | gain | chr8 | 1 | 146364022 |
| AOP01016C_WD | Follow-up |  | gain | chr9 | 1 | 141213431 |
| AOP01016C_WD | Follow-up |  | del | chr3 | 5510000 | 6140000 |
| AOP01017C_WD | Follow-up | 1 | del | chr3 | 114250000 | 114550000 |
| AOP01018A_WD | Follow-up | 2 | UPD | chr9 | 1 | 12790000 |
| AOP01018A_WD | Follow-up |  | UPD | chr9 | 1 | 32700000 |
| AOP01020B_WD | Follow-up | 0 |  |  |  |  |
| AOP01021A_WD | Follow-up | 4 | UPD | chr9 | 1 | 19440000 |
| AOP01021A_WD | Follow-up |  | UPD | chr19 | 1 | 19690000 |
| AOP01021A_WD | Follow-up |  | gain | chr1 | 223290000 | 223590000 |
| AOP01021A_WD | Follow-up |  | del | chr3 | 159900000 | 160000000 |
| AOP01022A_WD | Follow-up | 1 | UPD | chr9 | 1 | 33100000 |
| AOP02001L_WD | Follow-up | 1 | gain | chr1 | 76130000 | 76730000 |
| AOP02002N_WD | Follow-up | 0 |  |  |  |  |
| AOP02003A_GD | Follow-up | 2 | UPD | chr9 | 1 | 35480000 |
| AOP02003A_GD | Follow-up |  | UPD | chr9 | 1 | 49000000 |
| AOP02005I_WD | Follow-up | 0 |  |  |  |  |
| AOP03001I_WD | Follow-up | 1 | gain | chr20 | 32810000 | 33080000 |
| AOP03002H_WD | Follow-up | 0 |  |  |  |  |
| AOP03003C_GD | Follow-up | 2 | gain | chr3 | 180600000 | 180660000 |
| AOP03003C_GD | Follow-up |  | del | chr7 | 8560000 | 8630000 |
| AOP03004B_WD | Follow-up | 0 |  |  |  |  |
| AOP03005F_WD | Follow-up | 3 | UPD | chr9 | 1 | 35640000 |
| AOP03005F_WD | Follow-up |  | del | chr4 | 165060000 | 165160000 |
| AOP03005F_WD | Follow-up |  | del | chr17 | 33670000 | 33770000 |
| AOP03006C_WD | Follow-up | 1 | UPD | chr9 | 1 | 49000000 |
| AOP03007A_WD | Follow-up | 0 |  |  |  |  |
| AOP04010A_WD | Follow-up | 0 |  |  |  |  |
| AOP05001D_GD | Follow-up | 0 |  |  |  |  |
| AOP05002P_WD | Follow-up | 2 | UPD | chr9 | 1 | 28200000 |
| AOP05002P_WD | Follow-up |  | del | chr3 | 48010000 | 48090000 |
| AOP05003H_WD | Follow-up | 0 |  |  |  |  |
| AOP05004L_WD | Follow-up | 0 |  |  |  |  |
| AOP05005L_WD | Follow-up | 0 |  |  |  |  |
| AOP05007H_WD | Follow-up | 2 | UPD | chr9 | 1 | 36730000 |
| AOP05007H_WD | Follow-up |  | del | chrY | 1 | 59373566 |
| AOP05008G_WD | Follow-up | 0 |  |  |  |  |
| AOP05010E_WD | Follow-up | 0 |  |  |  |  |
| AOP05011D_WD | Follow-up | 1 | UPD | chr9 | 1 | 28790000 |
| AOP05012B_WD | Follow-up | 1 | gain | chr12 | 103750000 | 104330000 |
| AOP06001J_WD | Follow-up | 2 | UPD | chr9 | 1 | 28210000 |
| AOP06001J_WD | Follow-up |  | UPD | chr14 | 81790000 | 107349540 |
| AOP06003J_WD | Follow-up | 1 | UPD | chr9 | 1 | 20480000 |
| AOP06004F_WD | Follow-up | 0 |  |  |  |  |
| AOP06005F_WD | Follow-up | 1 | UPD | chr9 | 1 | 49000000 |
| AOP06006E_WD | Follow-up | 1 | UPD | chr9 | 1 | 16000000 |
| Baseline, peg-proline-IFNα-2b treatment start; UPD, uniparental disomy; del, deletion; coordinates based on hg19; | | | | | | |

| **Supporting Information Table III. *JAK2* mutant allele burden and molecular responses over time in PV patients treated with peg-proline-IFNα-2b.** | | | | | |
| --- | --- | --- | --- | --- | --- |
|  |  |  |  |  |  |
| **Sample ID** | **Sampling point** | **Time from baseline, days** | **Type of *JAK2* mutation** | ***JAK2* mutant allele burden** | **Molecular response** |
| AOP_01001_GD | Baseline | 0 | V617F | 42.00% |  |
| AOP01001A_GD | Follow-up | 92 | V617F | 40.00% | NMR |
| AOP01001B_GD | Follow-up | 147 | V617F | 37.00% | NMR |
| AOP01001C_GD | Follow-up | 203 | V617F | 46.00% | NMR |
| AOP01001D_GD | Follow-up | 268 | V617F | 25.00% | NMR |
| AOP01001F_GD | Follow-up | 396 | V617F | 39.00% | NMR |
| AOP01001G_GD | Follow-up | 450 | V617F | 42.00% | NMR |
| AOP01001H_GD | Follow-up | 512 | V617F | 42.00% | NMR |
| AOP01001I_WD | Follow-up | 631 | V617F | 39.00% | NMR |
| AOP01001J_WD | Follow-up | 735 | V617F | 29.00% | NMR |
| AOP01001K_WD | Follow-up | 844 | V617F | 31.00% | NMR |
| AOP01001L_WD | Follow-up | 959 | V617F | 27.00% | NMR |
| AOP01001M_WD | Follow-up | 1068 | V617F | 28.50% | NMR |
| AOP_01002_GD | Baseline | 0 | V617F | 86.00% |  |
| AOP01002A_GD | Follow-up | 83 | V617F | 89.00% | NMR |
| AOP01002B_GD | Follow-up | 138 | V617F | 90.00% | NMR |
| AOP01002C_GD | Follow-up | 194 | V617F | 93.00% | NMR |
| AOP01002D_GD | Follow-up | 259 | V617F | 86.00% | NMR |
| AOP01002E_GD | Follow-up | 317 | V617F | 89.00% | NMR |
| AOP01002F_GD | Follow-up | 387 | V617F | 92.00% | NMR |
| AOP01002G_GD | Follow-up | 441 | V617F | 86.00% | NMR |
| AOP01002H_GD | Follow-up | 503 | V617F | 99.00% | NMR |
| AOP01002I_WD | Follow-up | 614 | V617F | 88.00% | NMR |
| AOP01002J_WD | Follow-up | 726 | V617F | 81.00% | NMR |
| AOP01002K_WD | Follow-up | 835 | V617F | 72.00% | NMR |
| AOP01002L_WD | Follow-up | 950 | V617F | 80.00% | NMR |
| AOP01002M_WD | Follow-up | 1070 | V617F | 78.00% | NMR |
| AOP01002N_WD | Follow-up | 1127 | V617F | 76.00% | NMR |
| AOP_01003_GD | Baseline | 0 | V617F | 68.50% |  |
| AOP01003A_GD | Follow-up | 80 | V617F | 67.00% | NMR |
| AOP01003B_GD | Follow-up | 141 | V617F | 77.50% | NMR |
| AOP_01004_GD | Baseline | 0 | V617F | 23.50% |  |
| AOP01004A_GD | Follow-up | 80 | V617F | 37.00% | NMR |
| AOP01004B_GD | Follow-up | 140 | V617F | 72.00% | NMR |
| AOP01004D_GD | Follow-up | 259 | V617F | 23.00% | NMR |
| AOP01004E_GD | Follow-up | 316 | V617F | 27.00% | NMR |
| AOP01004F_GD | Follow-up | 371 | V617F | 25.00% | NMR |
| AOP01004G_GD | Follow-up | 427 | V617F | 29.00% | NMR |
| AOP01004H_GD | Follow-up | 486 | V617F | 36.00% | NMR |
| AOP01004I_WD | Follow-up | 595 | V617F | 30.00% | NMR |
| AOP01004J_WD | Follow-up | 707 | V617F | 23.00% | NMR |
| AOP01004K_WD | Follow-up | 833 | V617F | 25.00% | NMR |
| AOP01004L_WD | Follow-up | 959 | V617F | 25.25% | NMR |
| AOP01004M_WD | Follow-up | 1072 | V617F | 19.50% | NMR |
| AOP_01005_GD | Baseline | 0 | V617F | 43.00% |  |
| AOP01005A_GD | Follow-up | 79 | V617F | 56.00% | NMR |
| AOP01005B_GD | Follow-up | 142 | V617F | 36.00% | NMR |
| AOP_01009_GD | Baseline | 0 | V617F | 98.50% |  |
| AOP01009A_GD | Follow-up | 76 | V617F | 97.00% | NMR |
| AOP01009B_GD | Follow-up | 141 | V617F | 95.00% | NMR |
| AOP01009C_GD | Follow-up | 210 | V617F | 94.00% | NMR |
| AOP01009D_GD | Follow-up | 259 | V617F | 87.00% | NMR |
| AOP01009E_GD | Follow-up | 316 | V617F | 87.00% | NMR |
| AOP01009F_GD | Follow-up | 372 | V617F | 88.00% | NMR |
| AOP01009G_WD | Follow-up | 483 | V617F | 29.00% | PMR |
| AOP01009H_WD | Follow-up | 610 | V617F | 14.00% | PMR |
| AOP01009I_WD | Follow-up | 721 | V617F | 7.00% | PMR |
| AOP01009J_WD | Follow-up | 833 | V617F | 9.00% | PMR |
| AOP01009K_WD | Follow-up | 937 | V617F | 4.00% | PMR |
| AOP01009L_WD | Follow-up | 1057 | V617F | 3.00% | PMR |
| AOP_01011_GD | Baseline | 0 | V617F | 89.50% |  |
| AOP01011A_GD | Follow-up | 92 | V617F | 76.00% | NMR |
| AOP01011C_GD | Follow-up | 220 | V617F | 56.00% | NMR |
| AOP01011D_GD | Follow-up | 283 | V617F | 62.00% | NMR |
| AOP01011E_GD | Follow-up | 343 | V617F | 50.00% | NMR |
| AOP01011F_GD | Follow-up | 402 | V617F | 61.00% | NMR |
| AOP01011G_WD | Follow-up | 512 | V617F | 19.00% | PMR |
| AOP01011H_WD | Follow-up | 637 | V617F | 18.00% | PMR |
| AOP01011I_WD | Follow-up | 738 | V617F | 16.00% | PMR |
| AOP01011J_WD | Follow-up | 861 | V617F | 12.00% | PMR |
| AOP_01012_GD | Baseline | 0 | V617F | 42.50% |  |
| AOP01012A_GD | Follow-up | 15 | V617F | 62.00% | NMR |
| AOP01012B_GD | Follow-up | 84 | V617F | 26.00% | NMR |
| AOP01012C_WD | Follow-up | 140 | V617F | 50.00% | NMR |
| AOP01012D_WD | Follow-up | 267 | V617F | 27.00% | NMR |
| AOP01012E_WD | Follow-up | 308 | V617F | 26.50% | NMR |
| AOP_01013_WD | Baseline | 0 | V617F | 23.33% |  |
| AOP01013A_WD | Follow-up | 131 | V617F | 51.00% | NMR |
| AOP01013B_WD | Follow-up | 201 | V617F | 42.33% | NMR |
| AOP_01014_GD | Baseline | 0 | V617F | 68.00% |  |
| AOP01014A_WD | Follow-up | 153 | V617F | 35.00% | NMR |
| AOP01014B_WD | Follow-up | 286 | V617F | 33.00% | PMR |
| AOP01014C_WD | Follow-up | 406 | V617F | 35.50% | NMR |
| AOP01014D_WD | Follow-up | 616 | V617F | 32.50% | PMR |
| AOP_01015_WD | Baseline | 0 | V617F | 91.50% |  |
| AOP01015A_WD | Follow-up | 166 | V617F | 88.00% | NMR |
| AOP01015B_WD | Follow-up | 292 | V617F | 77.00% | NMR |
| AOP01015C_WD | Follow-up | 418 | V617F | 69.50% | NMR |
| AOP01015D_WD | Follow-up | 549 | V617F | 57.00% | NMR |
| AOP01015E_WD | Follow-up | 564 | V617F | 62.50% | NMR |
| AOP_01018_WD.1 | Baseline | 0 | V617F | 56.00% |  |
| AOP01018A_WD | Follow-up | 142 | V617F | 63.50% | NMR |
| AOP01018B_WD | Follow-up | 248 | V617F | 61.00% | NMR |
| AOP01018C_WD | Follow-up | 311 | V617F | 56.00% | NMR |
| AOP01018D_WD | Follow-up | 377 | V617F | 57.00% | NMR |
| AOP_01020_WD | Baseline | 0 | exon 12 | 25.00% |  |
| AOP01020A_WD | Follow-up | 135 | exon 12 | 20.00% | NMR |
| AOP01020B_WD | Follow-up | 230 | exon 12 | 20.50% | NMR |
| AOP_01021_WD | Baseline | 0 | V617F | 21.50% |  |
| AOP01021A_WD | Follow-up | 142 | V617F | 18.00% | NMR |
| AOP01021B_WD | Follow-up | 246 | V617F | 16.00% | NMR |
| AOP01021C_WD | Follow-up | 314 | V617F | 26.00% | NMR |
| AOP01021D_WD | Follow-up | 371 | V617F | 24.00% | NMR |
| AOP_01022_WD | Baseline | 0 | V617F | 71.50% |  |
| AOP01022A_WD | Follow-up | 133 | V617F | 40.00% | NMR |
| AOP01022B_WD | Follow-up | 299 | V617F | 61.00% | NMR |
| AOP01022C_WD | Follow-up | 362 | V617F | 28.50% | PMR |
| AOP_02001_GD | Baseline | 0 | V617F | 30.50% |  |
| AOP02001_WD | Follow-up | 90 | V617F | 32.00% | NMR |
| AOP02001A_GD | Follow-up | 117 | V617F | 26.00% | NMR |
| AOP02001B_GD | Follow-up | 145 | V617F | 38.00% | NMR |
| AOP02001C_GD | Follow-up | 202 | V617F | 34.00% | NMR |
| AOP02001D_GD | Follow-up | 266 | V617F | 18.00% | NMR |
| AOP02001E_GD | Follow-up | 342 | V617F | 22.00% | NMR |
| AOP02001F_GD | Follow-up | 412 | V617F | 23.00% | NMR |
| AOP02001G_GD | Follow-up | 440 | V617F | 16.00% | NMR |
| AOP02001H_GD | Follow-up | 455 | V617F | 16.00% | NMR |
| AOP02001I_GD | Follow-up | 505 | V617F | 18.00% | NMR |
| AOP02001J_WD | Follow-up | 559 | V617F | 15.00% | PMR |
| AOP02001K_WD | Follow-up | 616 | V617F | 13.00% | PMR |
| AOP02001L_WD | Follow-up | 750 | V617F | 9.00% | PMR |
| AOP02001M_WD | Follow-up | 974 | V617F | 5.00% | PMR |
| AOP02001N_WD | Follow-up | 1002 | V617F | 7.00% | PMR |
| AOP02001O_WD | Follow-up | 1126 | V617F | 6.00% | PMR |
| AOP_02002_GD | Baseline | 0 | V617F | 93.00% |  |
| AOP02002A_GD | Follow-up | 83 | V617F | 96.00% | NMR |
| AOP02002B_GD | Follow-up | 139 | V617F | 95.00% | NMR |
| AOP02002C_GD | Follow-up | 195 | V617F | 95.00% | NMR |
| AOP02002D_GD | Follow-up | 272 | V617F | 91.00% | NMR |
| AOP02002E_GD | Follow-up | 371 | V617F | 80.00% | NMR |
| AOP02002F_GD | Follow-up | 426 | V617F | 63.00% | NMR |
| AOP02002G_GD | Follow-up | 440 | V617F | 61.00% | NMR |
| AOP02002I_GD | Follow-up | 496 | V617F | 51.00% | NMR |
| AOP02002J_GD | Follow-up | 539 | V617F | 38.00% | PMR |
| AOP02002K_WD | Follow-up | 595 | V617F | 22.00% | PMR |
| AOP02002L_WD | Follow-up | 728 | V617F | 6.00% | PMR |
| AOP02002M_WD | Follow-up | 834 | V617F | 6.00% | PMR |
| AOP02002N_WD | Follow-up | 986 | V617F | 1.50% | PMR |
| AOP02002O_WD | Follow-up | 1098 | V617F | 1.50% | PMR |
| AOP_02003_GD | Baseline | 0 | V617F | 99.00% |  |
| AOP02003A_GD | Follow-up | 61 | V617F | 98.00% | NMR |
| AOP_03001_GD | Baseline | 0 | V617F | 45.50% |  |
| AOP03001A_GD | Follow-up | 82 | V617F | 55.00% | NMR |
| AOP03001B_GD | Follow-up | 140 | V617F | 36.00% | NMR |
| AOP03001C_GD | Follow-up | 202 | V617F | 57.00% | NMR |
| AOP03001D_GD | Follow-up | 306 | V617F | 40.00% | NMR |
| AOP03001F_WD | Follow-up | 383 | V617F | 20.00% | PMR |
| AOP03001G_WD | Follow-up | 509 | V617F | 4.00% | PMR |
| AOP03001H_WD | Follow-up | 678 | V617F | 0.00% | CMR |
| AOP03001I_WD | Follow-up | 797 | V617F | 2.50% | PMR |
| AOP03001J_WD | Follow-up | 918 | V617F | 0.00% | CMR |
| AOP_03005_GD | Baseline | 0 | V617F | 86.00% |  |
| AOP03005A_GD | Follow-up | 8 | V617F | 82.00% | NMR |
| AOP03005B_WD | Follow-up | 134 | V617F | 43.00% | PMR |
| AOP03005C_WD | Follow-up | 259 | V617F | 48.00% | NMR |
| AOP03005D_WD | Follow-up | 273 | V617F | 41.00% | PMR |
| AOP03005E_WD | Follow-up | 353 | V617F | 27.00% | PMR |
| AOP03005F_WD | Follow-up | 463 | V617F | 29.00% | PMR |
| AOP03005G_WD | Follow-up | 575 | V617F | 27.00% | PMR |
| AOP03005H_WD | Follow-up | 687 | V617F | 21.50% | PMR |
| AOP_03006_GD | Baseline | 0 | V617F | 32.00% |  |
| AOP03006A_WD | Follow-up | 133 | V617F | 10.00% | PMR |
| AOP03006B_WD | Follow-up | 245 | V617F | 3.00% | PMR |
| AOP03006C_WD | Follow-up | 353 | V617F | 12.50% | PMR |
| AOP_03007_WD | Baseline | 0 | V617F | 25.00% |  |
| AOP03007A_WD | Follow-up | 76 | V617F | 22.50% | NMR |
| AOP_04007_WD | Baseline | 0 | V617F | 48.00% |  |
| AOP04007A_WD | Follow-up | 255 | V617F | 38.00% | NMR |
| AOP_04010_WD | Baseline | 0 | V617F | 71.50% |  |
| AOP04010A_WD | Follow-up | 188 | V617F | 1.33% | PMR |
| AOP_05002_GD | Baseline | 0 | V617F | 73.00% |  |
| AOP05002A_GD | Follow-up | 78 | V617F | 78.00% | NMR |
| AOP05002B_GD | Follow-up | 141 | V617F | 91.00% | NMR |
| AOP05002C_GD | Follow-up | 195 | V617F | 85.00% | NMR |
| AOP05002D_GD | Follow-up | 260 | V617F | 80.00% | NMR |
| AOP05002E_GD | Follow-up | 320 | V617F | 53.00% | NMR |
| AOP05002F_GD | Follow-up | 379 | V617F | 83.00% | NMR |
| AOP05002G_GD | Follow-up | 434 | V617F | 61.00% | NMR |
| AOP05002H_GD | Follow-up | 449 | V617F | 85.00% | NMR |
| AOP05002I_WD | Follow-up | 491 | V617F | 54.00% | NMR |
| AOP05002J_WD | Follow-up | 546 | V617F | 40.00% | NMR |
| AOP05002K_WD | Follow-up | 603 | V617F | 32.00% | PMR |
| AOP05002L_WD | Follow-up | 713 | V617F | 16.00% | PMR |
| AOP05002M_WD | Follow-up | 757 | V617F | 15.00% | PMR |
| AOP05002N_WD | Follow-up | 827 | V617F | 12.00% | PMR |
| AOP05002O_WD | Follow-up | 883 | V617F | 7.00% | PMR |
| AOP05002P_WD | Follow-up | 939 | V617F | 6.00% | PMR |
| AOP05002Q_WD | Follow-up | 1044 | V617F | 5.00% | PMR |
| AOP_05003_GD | Baseline | 0 | V617F | 21.50% |  |
| AOP05003A_GD | Follow-up | 134 | V617F | 41.00% | NMR |
| AOP05003B_GD | Follow-up | 213 | V617F | 21.00% | NMR |
| AOP05003D_GD | Follow-up | 402 | V617F | 36.00% | NMR |
| AOP05003E_GD | Follow-up | 457 | V617F | 25.00% | NMR |
| AOP05003F_WD | Follow-up | 500 | V617F | 17.00% | NMR |
| AOP05003G_WD | Follow-up | 556 | V617F | 15.00% | NMR |
| AOP05003H_WD | Follow-up | 633 | V617F | 5.00% | PMR |
| AOP_05005_GD | Baseline | 0 | V617F | 26.50% |  |
| AOP05005A_GD | Follow-up | 77 | V617F | 26.00% | NMR |
| AOP05005B_GD | Follow-up | 147 | V617F | 23.00% | NMR |
| AOP05005C_GD | Follow-up | 187 | V617F | 18.00% | NMR |
| AOP05005E_GD | Follow-up | 315 | V617F | 14.00% | NMR |
| AOP05005F_GD | Follow-up | 399 | V617F | 30.00% | NMR |
| AOP05005G_GD | Follow-up | 427 | V617F | 25.00% | NMR |
| AOP05005H_WD | Follow-up | 483 | V617F | 17.00% | NMR |
| AOP05005I_WD | Follow-up | 538 | V617F | 15.00% | NMR |
| AOP05005J_WD | Follow-up | 595 | V617F | 6.00% | PMR |
| AOP05005K_WD | Follow-up | 707 | V617F | 11.00% | PMR |
| AOP05005L_WD | Follow-up | 763 | V617F | 6.50% | PMR |
| AOP05005M_WD | Follow-up | 931 | V617F | 6.00% | PMR |
| AOP05005N_WD | Follow-up | 1043 | V617F | 4.00% | PMR |
| AOP_05007_GD | Baseline | 0 | V617F | 62.50% |  |
| AOP05007A_GD | Follow-up | 87 | V617F | 48.00% | NMR |
| AOP05007C_GD | Follow-up | 213 | V617F | 61.00% | NMR |
| AOP05007D_WD | Follow-up | 269 | V617F | 28.00% | PMR |
| AOP05007E_WD | Follow-up | 329 | V617F | 25.00% | PMR |
| AOP05007F_WD | Follow-up | 402 | V617F | 6.00% | PMR |
| AOP05007G_WD | Follow-up | 513 | V617F | 12.00% | PMR |
| AOP05007H_WD | Follow-up | 633 | V617F | 5.50% | PMR |
| AOP05007I_WD | Follow-up | 752 | V617F | 3.00% | PMR |
| AOP_05008_GD | Baseline | 0 | V617F | 40.50% |  |
| AOP05008A_WD | Follow-up | 130 | V617F | 30.00% | NMR |
| AOP05008B_WD | Follow-up | 171 | V617F | 26.00% | NMR |
| AOP05008C_WD | Follow-up | 241 | V617F | 21.00% | NMR |
| AOP05008D_WD | Follow-up | 297 | V617F | 21.00% | NMR |
| AOP05008E_WD | Follow-up | 353 | V617F | 25.00% | NMR |
| AOP05008F_WD | Follow-up | 409 | V617F | 25.00% | NMR |
| AOP05008G_WD | Follow-up | 500 | V617F | 17.50% | PMR |
| AOP_05010_WD | Baseline | 0 | V617F | 28.00% |  |
| AOP05010A_WD | Follow-up | 140 | V617F | 10.00% | PMR |
| AOP05010B_WD | Follow-up | 196 | V617F | 9.00% | PMR |
| AOP05010C_WD | Follow-up | 252 | V617F | 7.00% | PMR |
| AOP05010D_WD | Follow-up | 308 | V617F | 4.00% | PMR |
| AOP05010E_WD | Follow-up | 364 | V617F | 3.50% | PMR |
| AOP05010F_WD | Follow-up | 476 | V617F | 0.00% | CMR |
| AOP_05011_WD | Baseline | 0 | V617F | 37.00% |  |
| AOP05011A_WD | Follow-up | 127 | V617F | 22.00% | NMR |
| AOP05011B_WD | Follow-up | 197 | V617F | 22.00% | NMR |
| AOP05011C_WD | Follow-up | 253 | V617F | 14.00% | PMR |
| AOP05011D_WD | Follow-up | 309 | V617F | 8.50% | PMR |
| AOP05011E_WD | Follow-up | 351 | V617F | 8.00% | PMR |
| AOP05011F_WD | Follow-up | 365 | V617F | 6.00% | PMR |
| AOP05011G_WD | Follow-up | 477 | V617F | 6.00% | PMR |
| AOP_06001_GD | Baseline | 0 | V617F | 45.00% |  |
| AOP06001A_GD | Follow-up | 79 | V617F | 82.00% | NMR |
| AOP06001B_GD | Follow-up | 135 | V617F | 85.00% | NMR |
| AOP06001C_GD | Follow-up | 191 | V617F | 83.00% | NMR |
| AOP06001D_GD | Follow-up | 247 | V617F | 89.00% | NMR |
| AOP06001E_GD | Follow-up | 303 | V617F | 70.00% | NMR |
| AOP06001F_GD | Follow-up | 359 | V617F | 79.00% | NMR |
| AOP06001G_GD | Follow-up | 415 | V617F | 80.00% | NMR |
| AOP06001H_GD | Follow-up | 485 | V617F | 88.00% | NMR |
| AOP06001I_WD | Follow-up | 583 | V617F | 74.00% | NMR |
| AOP06001J_WD | Follow-up | 703 | V617F | 49.50% | NMR |
| AOP_06003_GD | Baseline | 0 | V617F | 80.50% |  |
| AOP06003A_GD | Follow-up | 92 | V617F | 88.00% | NMR |
| AOP06003B_GD | Follow-up | 147 | V617F | 90.00% | NMR |
| AOP06003C_GD | Follow-up | 210 | V617F | 66.00% | NMR |
| AOP06003D_GD | Follow-up | 266 | V617F | 58.00% | NMR |
| AOP06003E_GD | Follow-up | 322 | V617F | 44.00% | NMR |
| AOP06003F_GD | Follow-up | 402 | V617F | 80.00% | NMR |
| AOP06003G_WD | Follow-up | 490 | V617F | 53.00% | NMR |
| AOP06003H_WD | Follow-up | 602 | V617F | 17.00% | PMR |
| AOP06003I_WD | Follow-up | 715 | V617F | 26.00% | PMR |
| AOP06003J_WD | Follow-up | 843 | V617F | 20.00% | PMR |
| AOP06003K_WD | Follow-up | 954 | V617F | 14.00% | PMR |
| AOP06003L_WD | Follow-up | 1073 | V617F | 17.50% | PMR |
| AOP_06005_WD | Baseline | 0 | V617F | 63.50% |  |
| AOP06005A_GD | Follow-up | 76 | V617F | 82.00% | NMR |
| AOP06005B_WD | Follow-up | 132 | V617F | 93.00% | NMR |
| AOP06005C_WD | Follow-up | 251 | V617F | 82.00% | NMR |
| AOP06005D_WD | Follow-up | 364 | V617F | 85.00% | NMR |
| AOP06005E_WD | Follow-up | 473 | V617F | 83.00% | NMR |
| AOP06005F_WD | Follow-up | 601 | V617F | 80.50% | NMR |
| AOP06005G_WD | Follow-up | 713 | V617F | 52.50% | NMR |
| AOP_06006_WD | Baseline | 0 | V617F | 77.00% |  |
| AOP06006A_WD | Follow-up | 92 | V617F | 57.00% | NMR |
| AOP06006B_WD | Follow-up | 161 | V617F | 67.00% | NMR |
| AOP06006C_WD | Follow-up | 261 | V617F | 62.00% | NMR |
| AOP06006D_WD | Follow-up | 373 | V617F | 58.00% | NMR |
| AOP06006E_WD | Follow-up | 527 | V617F | 53.00% | NMR |
| AOP06006F_WD | Follow-up | 584 | V617F | 47.00% | NMR |
| baseline, peg-proline-IFNα-2b treatment start; CMR, complete molecular response; PMR, partial molecular response; NMR, no molecular response; | | | | | |

**
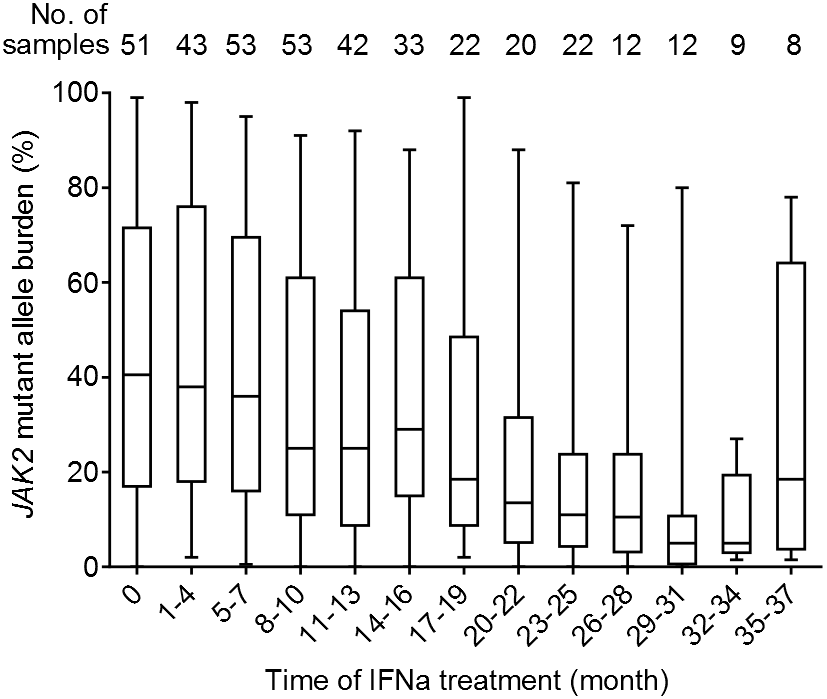
**

**Supporting Information Figure 1**. ***JAK2* mutant allele burden change over time for the complete cohort of PV patients treated with peg-proline-IFNα-2b.**

(0, baseline/ peg-proline-IFNa-2b treatment start; horizontal lines, median values; bars, minimum and maximum values; box, values between 25^th^ to 75^th^ percentile)
